# Suppression of SREBP by a transmembrane protein mutated in cardiomyopathy

**DOI:** 10.1016/j.jbc.2025.110644

**Published:** 2025-08-28

**Authors:** Yasushi Takemoto, Manchir Tserendagva, Motonari Uesugi

**Affiliations:** 1 Institute for Chemical Research, Kyoto University, Uji, Kyoto, Japan; 2 Institute for Integrated Cell-Material Sciences (WPI-iCeMS), Kyoto University, Uji, Kyoto, Japan; 3 School of Pharmacy, Fudan University, Shanghai, China

**Keywords:** SREBP, TMEM43, lipid synthesis, cardiomyopathy, adipogenesis, transcription regulation, transcription coactivator

## Abstract

Arrhythmogenic right ventricular cardiomyopathy (ARVC) is an inherited cardiac disorder characterized by the replacement of myocardial tissue with adipose tissue. One of the frequently mutated proteins in ARVC is TMEM43, which encodes a transmembrane protein primarily localized to the endoplasmic reticulum (ER) and nuclear membranes and stabilized by interacting with squalene synthase (SQS). However, its precise role in ARVC pathogenesis remains unclear. The present study demonstrates that TMEM43 suppresses the expression of SQS and other lipogenic proteins by inhibiting sterol regulatory element-binding proteins (SREBPs). Mechanistic analysis revealed that TMEM43 inhibits nuclear SREBP activity by binding to LRPPRC, which serves as a transcriptional coactivator for SREBP in coordination with PGC1β. TMEM43 sequesters LRPPRC to the nuclear membrane, disrupting its coactivator function and inhibiting SREBP activity. Given the role of SREBPs in adipogenesis, our findings highlight TMEM43 as a key regulator of adipocyte *versus* cardiomyocyte differentiation.

Arrhythmogenic right ventricular cardiomyopathy (ARVC) is an inherited cardiac disorder characterized by the substitution of myocardial tissue with adipose tissue (1). Recent studies utilizing the Clinical Genome Resource framework (2) have identified six genes—namely, *PKP2* (3), *DSP* (4), *DSG2* (5), *DSC2* (6), *JUP* (7), and *TMEM43* (8)—as being associated with ARVC. The five genes (*PKP2*, *DSP*, *DSG2*, *DSC2*, *JUP*) encode desmosome proteins important for cell-cell adhesion, expressed abundantly in the epidermis and myocardium. In contrast, transmembrane protein 43 (encoded by *TMEM43*) has emerged as one of the few non-desmosomal proteins associated with ARVC (8). A missense mutation, S358L, in *TMEM43* is linked to the development of arrhythmogenic right ventricular cardiomyopathy type 5 (ARVC5) (8), a highly penetrant and lethal type with right ventricular dilation, fibro-fatty replacement of cardiomyocytes, heart failure, and early death (9).

TMEM43 is a 4-times membrane protein that exhibits localization on the endoplasmic reticulum (ER) and nuclear membranes (10). Our previous investigations have revealed that TMEM43 interacts with squalene synthase (SQS) to achieve stabilization on the ER membrane (11). Disruption of this interaction, either through the S358 L clinical mutation of TMEM43 or treatment with KY02111, a small molecule modulator of cardiomyogenesis (12), results in the degradation of TMEM43, leading to the suppression of TGFβ signaling and the induction of adipogenesis during cardiac differentiation (11). Other research groups have also endeavored to elucidate the underlying mechanism by which TMEM43 leads to fibrosis in ARVC through modulation of TGFβ (13, 14). At the nuclear membrane, TMEM43 engages with lamin A, lamin B, emerin, and SUN domain-containing protein 2 (15), thereby playing a role in the maintenance of nuclear envelope structure (10, 16). In line with this nuclear-membrane function of TMEM43, ARVC5 is accompanied by an observed increase in cell nucleus stiffness (9). Nevertheless, the precise role of nuclear TMEM43 in the pathogenesis of cardiomyopathy remains to be fully elucidated.

Sterol regulatory element-binding proteins (SREBPs) represent transcription factors that control adipogenesis and lipogenesis (17, 18, 19, 20). Newly synthesized SREBPs initially reside on the ER membrane, where they form a complex with SREBP cleavage-activating protein (SCAP), a specific escort protein of SREBPs. Under conditions of cellular sterol deficiency, the SREBP-SCAP complex undergoes intracellular trafficking from the ER to the Golgi apparatus. Within the Golgi, SREBPs undergo sequential cleavage by site-1 protease (S1P) and site-2 protease (S2P), liberating the NH_2_-terminal transcription factor domain of SREBPs. Subsequently, the mature form of SREBPs translocate to the nucleus, where they activate lipogenic genes. The activation of SREBP is tightly regulated by a negative feedback loop. This mechanism involves the binding of 25-hydroxycholesterol (25-HC) and cholesterol to insulin-induced genes (INSIGs) and to SCAP, respectively. This interaction prompts the association of SCAP with INSIGs, ultimately leading to the retention of the SREBP-SCAP complex within the ER (21).

The present study elucidates the involvement of nuclear membrane-localized TMEM43 in the suppression of SREBP-mediated gene activation. While exploring the interplay between TMEM43 and SQS, we serendipitously observed that TMEM43 downregulates the expression of *SQS*, a gene under the regulatory control of SREBP2. Utilizing this observation as a foundation, we embarked on a series of biochemical and cell biological experiments, ultimately unveiling an unprecedented mode of SREBP suppression mediated by TMEM43.

## Results

### TMEM43 suppresses SQS expression by attenuating SREBP activity

**Figure 1:**
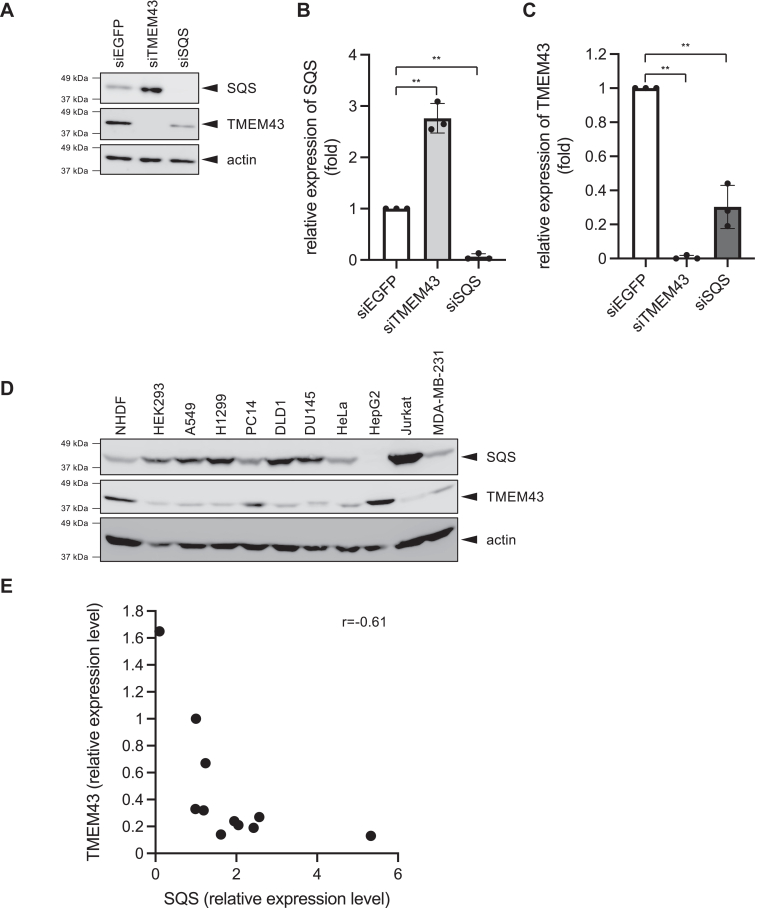
**TMEM43 suppresses SQS expression**. *A–C*, TMEM43 knockdown increases SQS expression. A549 cells were transfected with each siRNA (siEGFP, siSQS, or siTMEM43). After 4 days, the lysates were analyzed by western blotting (*A*). Expression levels of SQS (*B*) and TMEM43 (*C*) were analyzed by ImageQuant (GE Healthcare) (n = 3). Data are presented as mean of ± SD. Statistical significance was determined by paired two-tailed Student's *t* test. ∗*p* < 0.05, ∗∗*p* < 0.01. *D and E*, inverse correlation between TMEM43 expression and SQS expression. *D*, Western blotting analysis of SQS, TMEM43 and actin expressions in various cells. *E*, quantitation of expression levels of SQS and TMEM43 was analyzed by ImageQuant. Simple linear correlations between two parameters were calculated. Correlation coefficients (r) are shown in this figure. Similar results were obtained from three independent experiments.

Our prior investigations have demonstrated the interaction between TMEM43 and SQS and its role in stabilizing TMEM43 on the endoplasmic reticulum (ER) membrane (11). In fact, siRNA knockdown of SQS reduced TMEM43 protein levels (Fig. 1, *A*–*C*). Unexpectedly, the knockdown of TMEM43 led to an elevation in SQS protein levels (Fig. 1, *A*–*C*). This intriguing observation prompted us to extend our study to assess the protein levels of SQS and TMEM43 across various cultured cell lines. The results demonstrate an inverse correlation in the expression levels of these two proteins, which cannot be explained by the SQS-mediated stabilization of TMEM43 (Fig. 1, *D* and *E*). These findings suggest that, in addition to SQS-mediated stabilization of TMEM43, there exists an as-yet-unidentified mechanism that regulates the balance between SQS and TMEM43.

**Figure 2:**
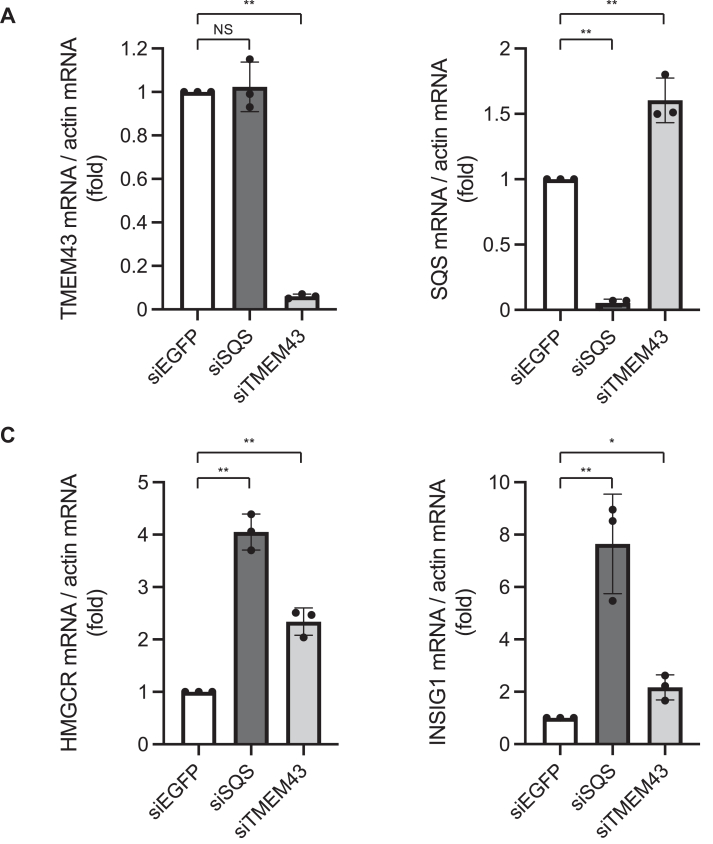
**TMEM43 suppresses SREBP-regulated gene expression**. TMEM43 knockdown increases SREBP-regulated gene expression. *A–D*, A549 cells were transfected with each siRNA (siEGFP, siSQS, or siTMEM43) and incubated for 4 days. The mRNA level of TMEM43 (*A*), SQS (*B*), HMGCR (*C*) or INSIG1 (*D*) was assessed by qPCR analysis. Data were analyzed by ΔΔCt method with actin as reference control (n = 3). Data are presented as mean of ± SD. Statistical significance was determined by paired two-tailed Student's *t* test. ∗*p* < 0.05, ∗∗*p* < 0.01. NS: not significant.

Intrigued by these observed protein-level interactions, we further investigated the mRNA levels of SQS and TMEM43 following their respective knockdowns (Fig. 2, *A* and *B*). The results revealed that the silencing of TMEM43 resulted in a marked increase in SQS mRNA levels, while the knockdown of SQS exhibited no appreciable effect on the mRNA expression of TMEM43. These findings collectively suggest that TMEM43 suppresses the SQS expression at a transcriptional level. We note that the increase in SQS protein levels upon TMEM43 knockdown (Fig. 1*A*) appears more pronounced than the corresponding increase in SQS mRNA levels (Fig. 2*B*). This raises the possibility that TMEM43 may regulate SQS expression not only at the transcriptional level through SREBPs but also through unknown mechanisms affecting protein turnover.

The transcription of *SQS* is known to be controlled by SREBPs (22, 23). Indeed, the SQS expression was increased in response to lipid depletion, a typical condition known to activate SREBPs. The induction of SQS expression under lipid-depleted conditions was mitigated by overexpressing TMEM43 (Fig. S1). These observations led us to postulate that TMEM43 downregulates the activity of SREBPs, thereby reducing SQS expression. To test the hypothesis, we initiated an assessment of the impact of TMEM43 knockdown on the mRNA levels of representative genes under the regulation of SREBPs. Among these genes, *HMGCR* and *INSIG1* are widely acknowledged as prominent targets of SREBPs (24, 25). As shown in Figure 2, *C* and *D*, the knockdown of SQS, a key enzyme for cholesterol biosynthesis (26, 27), activated SREBPs by lowering cellular cholesterol levels, thereby increasing HMGCR and INSIG1 mRNA levels. Importantly, TMEM43 knockdown also increased HMGCR and INSIG1 mRNA levels. These findings collectively suggest the role of TMEM43 in the suppression of SREBP-mediated gene expression.

**Figure 3:**
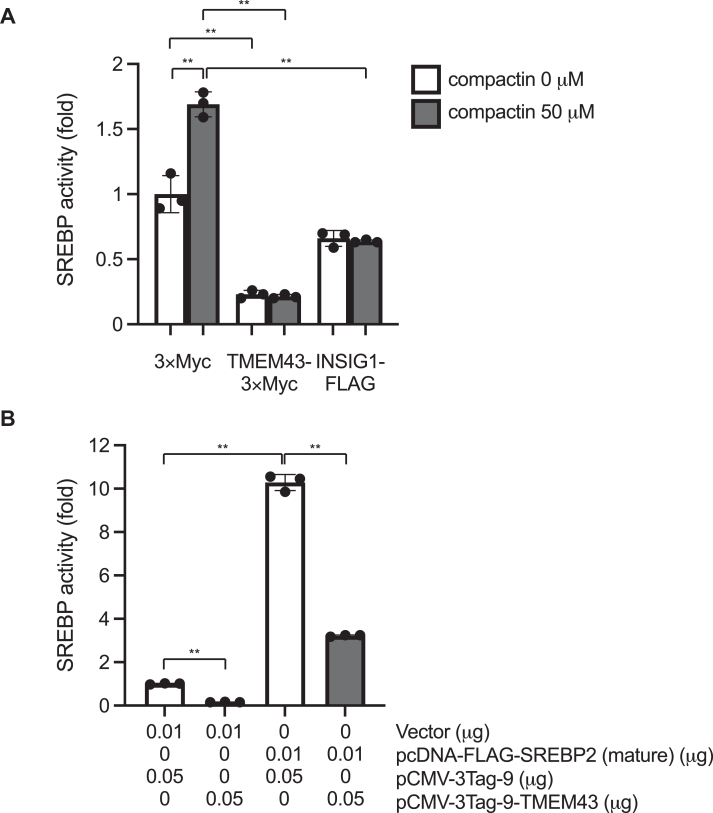
**TMEM43 suppresses SREBP transcriptional activity**. Effect of TMEM43 on SREBP transcriptional activity. *A*, HEK293 cells were transfected with pCMV-3Tag-9 (0.05 μg), pCMV-3Tag-9-TMEM43 (0.05 μg) or pCMV-INSIG1-FLAG (0.05 μg) together with pSRE-Luc (0.045 μg) and pAc-β-gal (0.005 μg) and incubated for 24 h. Then, DMSO or compactin (50 μM) was treated. After 24 h, luciferase activity was measured (n = 3). Data are presented as mean of ± SD. Statistical significance was determined by paired two-tailed Student's *t* test. ∗∗*p* < 0.01. Similar results were obtained from two independent experiments. *B*, HEK293 cells were transfected with pCMV-3Tag-9 (0.05 μg) or pCMV-3Tag-9-TMEM43 (0.05 μg), pcDNA-FLAG-SREBP2 (mature) (0.01 μg) or vector control (0.01 μg) together with pSRE-Luc (0.035 μg) and pAc-β-gal (0.005 μg). After 48 h, luciferase activity was measured (n = 3). Data are presented as mean of ± SD. Statistical significance was determined by paired two-tailed Student's *t* test. ∗∗*p* < 0.01. Similar results were obtained from two independent experiments.

To corroborate the inhibitory effect of TMEM43 on SREBPs, we employed a luciferase reporter gene under the regulation of three repeats of SREBP-binding sites. As anticipated, the activation of the SREBP reporter gene was induced by the addition of compactin (28, 29, 30), an inhibitor of HMGCR known to lower cellular cholesterol levels (Fig. 3*A*). We further validated the SREBP-responsiveness of the reporter by observing that both the compactin-induced SREBP activation and the basal SREBP activity were repressed upon overexpression of INSIG1, a well-recognized inhibitory protein of SREBPs. Notably, the overexpression of TMEM43 exhibited even greater potency in inhibiting SREBPs than that of INSIG1. In contrast, overexpression of TMEM43 displayed no detectable effects on a CMV promoter-controlled luciferase reporter gene (Fig. S2). These results collectively support the notion that TMEM43 suppresses the expression of SREBP-regulated genes by attenuating SREBPs.

### TMEM43 inhibits the nuclear, mature form of SREBP

SREBPs, initially synthesized as membrane-anchored precursors within the endoplasmic reticulum (ER), undergo proteolytic cleavage facilitated by two proteases (S1P and S2P) anchored to the Golgi membrane. This processing liberates a mature form of SREBPs from the Golgi membrane to the nucleus, leading to transcriptional activation of target genes (17, 19). To gain insights into the underlying mechanism of the TMEM43-mediated suppression of SREBPs, we exogenously expressed the mature form of SREBP2 (amino acids 1–481), which bypasses the requirement for the conventional proteolytic activation process at the Golgi, and analyzed its ability as a transcriptional activator in the presence of TMEM43. As shown in Figure 3*B*, expression of FLAG-tagged mature SREBP2 robustly activated the SRE promoter. This activation was canceled upon co-expression of TMEM43-3 × Myc, indicating that TMEM43 inhibits the activity of nuclear, mature SREBP2.

SREBP2 is primarily responsible for regulating cholesterol synthesis, while SREBP1a and SREBP1c are key regulators of fatty acid synthesis (19, 31). In line with the effect on SREBP2, we confirmed that TMEM43-3 × Myc also suppressed SRE promoter activation by mature SREBP1a (amino acids 1–490) (Fig. S3*A*). Furthermore, knockdown of TMEM43 led to increased gene expression of fatty acid synthase (FAS), a major SREBP1-target gene involved in fatty acid synthesis (32) (Fig. S3, *B* and *C*). These results collectively provide evidence that TMEM43 negatively regulates the transcriptional activity of nuclear, mature forms of both SREBP2 and SREBP1a.

TMEM43's subcellular localization has previously been documented to encompass both the endoplasmic reticulum (ER) and nuclear membrane (10). In our own investigations, we corroborated this localization pattern by detecting EGFP-fused TMEM43 along the nuclear membrane co-localized with NUP98, a component of the nuclear pore complex, as well as in close proximity to the nuclear region, conceivably corresponding to the ER co-localized with CANX, which localizes in ER (Fig. S4, *A* and *B*). The ability of EGFP-fused TMEM43 to suppress SREBP activation was confirmed by a luciferase reporter assay under a lipid-depleted condition (Fig. S5). The substantial localization of TMEM43 within the nuclear membrane prompted us to examine whether TMEM43 affects the nuclear translocation of the mature form of SREBP2. To address this question, HEK293 cells expressing FLAG-SREBP2 (mature) with TMEM43-3 × Myc were lysed and fractionated into the nuclear and cytosolic fractions, followed by Western blot analysis (Fig. S6*A*). Our fractionation protocol successfully distinguished between the nuclear and the cytosolic fractions, as evident from the predominant presence of α-tubulin, a cytosolic protein, in the cytosolic fraction, and NUP98, a nuclear protein, in the nuclear fraction. Moreover, the ER protein CANX was predominantly detected in the cytosolic fraction, validating the presence of ER in this fraction. As expected, TMEM43-3 × Myc was detected in both the nuclear and cytosolic fractions. FLAG-SREBP2 (mature) was primarily detected within the nuclear fraction, irrespective of the presence or absence of TMEM43-3 × Myc, indicating that TMEM43 has no detectable effect on the nuclear translocation of the mature form of SREBP2.

### TMEM43 suppresses LRPPRC/PGC1β-mediated SREBP activation

**Figure 4:**
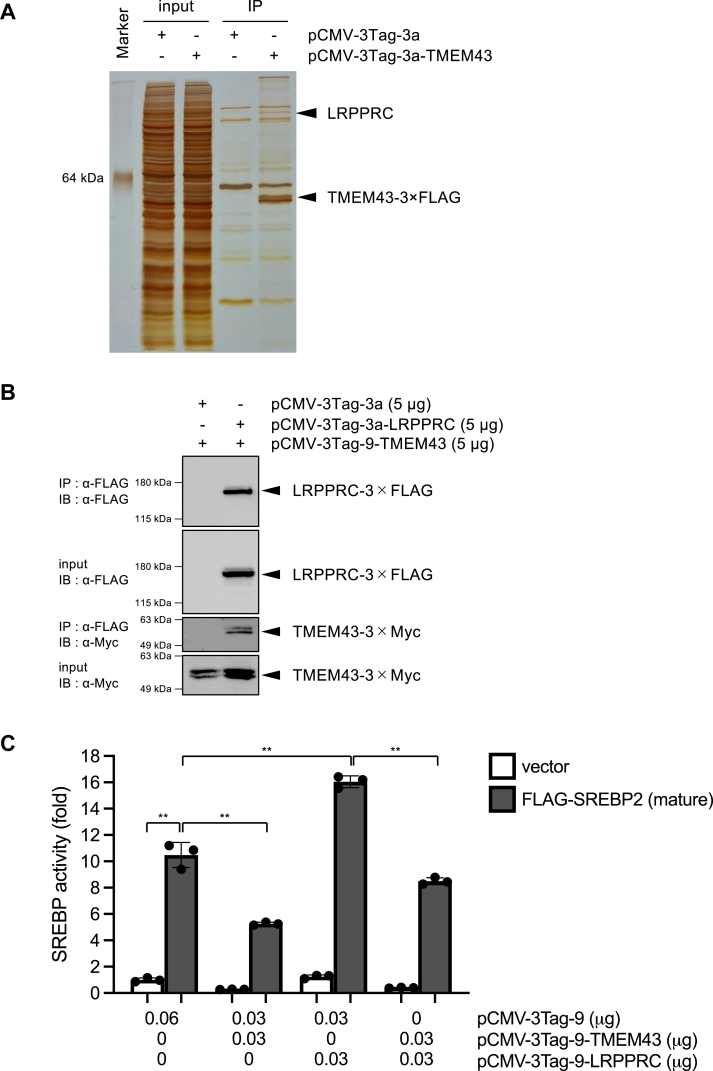
**TMEM43 suppresses LRPPRC-mediated SREBP activation.***A*, identification of LRPPRC as a binding protein of TMEM43. HEK293 cells were expressed with 3×FLAG or TMEM43-3 × FLAG. After immunoprecipitation with α–FLAG antibody, eluted proteins were detected by silver stain. LRPPRC was identified by mass analysis. *B*, HEK293 cells were expressed with 3×FLAG or LRPPRC-3 × FLAG and TMEM43-3 × Myc. After immunoprecipitation with α–FLAG antibody, eluted proteins were analyzed by western blotting. Similar results were obtained from two independent experiments. *C*, HEK293 cells were transfected with pCMV-3Tag-9, pCMV-3Tag-9-TMEM43 and/or pCMV-3Tag9-LRPPRC, pcDNA-FLAG-SREBP2 (mature) (0.01 μg) or vector control (0.01 μg) together with pSRE-Luc (0.025 μg) and pAc-β-gal (0.005 μg). After 48 h, luciferase activity was measured (n = 3). Data are presented as mean of ± SD. Statistical significance was determined by paired two-tailed Student's *t* test. ∗∗*p* < 0.01. Similar results were obtained from two independent experiments.

One possible mechanism underlying TMEM43-mediated inhibition of SREBPs could involve a direct physical interaction between these two proteins. However, our immunoprecipitation experiments indicated that TMEM43 does not display any detectable interaction with the mature form of SREBP2, implying that TMEM43 modulates SREBPs in an indirect manner (Fig. S6*B*). We therefore set out to identify TMEM43-binding proteins from proteomic lysates of HEK293 cells expressing TMEM43-3 × FLAG. These lysates were subjected to immunoprecipitation with an α-FLAG antibody, followed by SDS-PAGE and silver staining. This analysis revealed a single band that is selectively associated with TMEM43-3 × FLAG (Fig. 4*A*). LC-MS/MS analysis of this band unveiled the identity of the interacting protein as Leucine-rich PPR-motif-containing protein (LRPPRC), which predominantly localizes within the nucleus and mitochondria (33). Subsequently, the identity of the band was confirmed by western blotting (Fig. 4*B*). Fluorescent immunostaining also showed co-localization of EGFP-fused TMEM43 and LRPPRC at the nuclear membrane (Fig. S7*A*) and TMEM43 knockdown altered the distribution of LRPPRC (Fig. S7*B*), suggesting that TMEM43 interacts with LRPPRC at the nuclear membrane.

LRPPRC, which belongs to the pentatricopeptide repeat (PPR) motif-containing proteins family, is a multifunctional protein involved in energy metabolism (34, 35, 36). We examined the LRPPRC impact over SREBP activity by luciferase reporter assays. As shown in Figure 4*C*, co-expression of LRPPRC-3 × Myc enhanced the ability of FLAG-SREBP2 (mature) to induce SRE-promoter activation, indicating that LRPPRC acts as a co-activator of SREBP. This LRPPRC-induced potentiation was canceled by co-expression of TMEM43-3 × Myc, consistent with the observed interaction of TMEM43 with LRPPRC.

**Figure 5:**
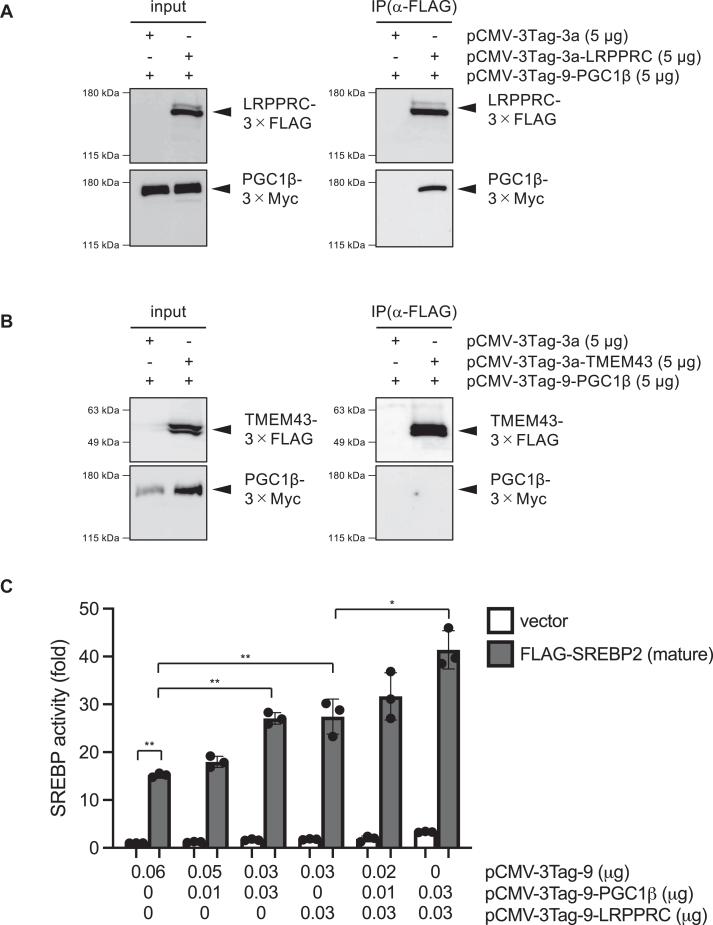
**LRPPRC and PGC1β additively activate SREBP transcriptional activity**. *A and B*, HEK293 cells were expressed with PGC1β-3 × Myc and 3 × FLAG or LRPPRC-3 × FLAG (*A*) or TMEM43-3 × FLAG (*B*). After immunoprecipitation with α–FLAG antibody, eluted proteins were analyzed by western blotting. Similar results were obtained from two independent experiments. *C*, HEK293 cells were transfected with pCMV-3Tag-9, pCMV-3Tag-9-PGC1β and/or pCMV-3Tag-9-LRPPRC, pcDNA-FLAG-SREBP2 (mature) (0.01 μg) or vector control (0.01 μg) together with pSRE-Luc (0.025 μg) and pAc-β-gal (0.005 μg). After 48 h, luciferase activity was measured (n = 3). Data are presented as mean of ± SD. Statistical significance was determined by paired two-tailed Student's *t* test. ∗*p* < 0.05, ∗∗*p* < 0.01. Similar results were obtained from two independent experiments.

LRPPRC has been identified as a binding partner of PGC1α, a transcriptional co-activator important in energy metabolism (37). The LRPPRC's binding capacity extends not only to PGC1α but also encompasses PGC1β, thereby enhancing their ability as co-activators. Given that PGC1β has been identified as a co-activator for SREBPs (38), our results prompted the hypothesis that LRPPRC might cooperate with PGC1β to stimulate SREBP activity. Indeed, our immunoprecipitation experiments confirmed the physical interaction between LRPPRC-3 × FLAG and PGC1β-3 × Myc (Fig. 5*A*). In contrast, no detectable physical interaction was observed between TMEM43-3 × FLAG and PGC1β-3 × Myc (Fig. 5*B*) and between LRPPRC-3 × Myc and 3 × FLAG-SREBP2(mature) (Fig. S8), suggesting that LRPPRC promotes SREBP2-mediated transcription indirectly, most likely through its interaction with PGC-1β. Note that co-expression of TMEM43-3 × FLAG led to an increase in PGC1β-3 × Myc protein levels through an as-yet-unknown mechanism, warranting further investigation.

To assess the impact of LRPPRC and PGC1β on SREBP activity, we conducted reporter gene experiments. As depicted in Figure 5*C*, the activation of the SRE promoter mediated by mature SREBP exhibited a dose-dependent increase upon the expression of PGC1β-3 × Myc. Co-expression of LRPPRC-3 × Myc and PGC1β-3 × Myc led to an additive enhancement of SREBP's transcriptional activity. These results collectively suggest that LRPPRC collaborates with PGC1β as co-activators of SREBP, with TMEM43 playing a pivotal role in binding to LRPPRC and subsequently suppressing LRPPRC/PGC1β-mediated SREBP activation.

### TMEM43 suppresses adipocyte differentiation

**Figure 6:**
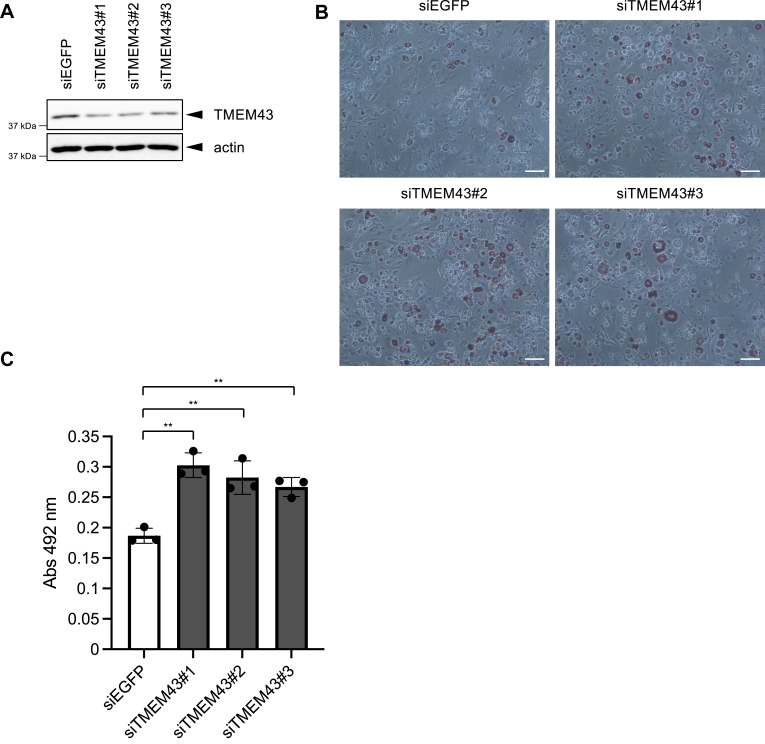
**TMEM43 suppresses adipocyte differentiation**. TMEM43 knockdown increases adipocyte differentiation. *A*, 3T3-L1 cells were transfected with each siRNA (siEGFP or siTMEM43#1, #2 or #3). After 2 days, the lysates were analyzed by western blotting. Similar results were obtained from two independent experiments. *B and C*, 3T3-L1 cells were transfected with each siRNA (siEGFP or siTMEM43#1, #2 or #3). After 2 days, the medium was replaced with MDI medium and incubated for 3 days. Then, the medium was replaced with insulin medium and incubated for 3 days. After then, the medium was replaced with normal medium. After 3 days of incubation, cells were stained with Oil Red O (*B*) (Scale bar: 100 μm). Oil Red O incorporated into cells was extracted with 2-propanol and the absorbance (492 nm) was measured (*C*) (n = 3). Data are presented as mean of ± SD. Statistical significance was determined by paired two-tailed Student's *t* test. ∗∗*p* < 0.01. Similar results were obtained from two independent experiments.

Given the well-established role of SREBPs in adipocyte differentiation (18, 20), we examined whether TMEM43 exerts any influence on this process. To investigate this, we employed 3T3-L1 cells, which can be differentiated into adipocytes upon stimulation with insulin, dexamethasone (a glucocorticoid receptor agonist), and isobutylmethylxanthine (a phosphodiesterase inhibitor) (39). Following adipocyte differentiation, the cells were subjected to Oil red O staining, a standard procedure for the identification of lipid-rich adipocyte cells. Upon the siRNA knockdown of TMEM43 (Fig. 6*A*), we observed a significant increase in Oil red O-positive cells (Fig. 6, *B* and *C*). This observed augmentation in Oil red O-positive cells serves as an indicator that the suppression of TMEM43 promotes adipocyte differentiation. Collectively, these findings suggest that TMEM43's inhibitory effect on SREBP activity leads to the suppression of adipocyte differentiation.

## Discussion

Mesodermal cells undergo differentiation into cardiomyocytes within the cardiac tissue. However, in the context of ARVC, an aberrant differentiation pattern emerges, diverting cells that would typically become cardiomyocytes towards adipogenic differentiation, ultimately contributing to the onset of fibrotic changes. The pivotal trigger for the pathogenesis of ARVC5 is attributed to the S358L mutation within TMEM43. This mutation has been documented to reduce the stability of TMEM43 and concomitantly elevate plasma cholesterol levels (40, 41, 42). We overexpressed the TMEM43 S358L mutant and examined its effect on SREBP activity. Similar to the wild-type (WT) protein, the S358L mutant suppressed SREBP activity, suggesting that this mutation had no significant impact on TMEM43's ability to inhibit SREBP (Fig. S9*A*). However, cycloheximide chase experiments revealed that the S358L mutant is less stable than the WT protein, consistent with our previous observation (Fig. S9, *B* and *C*). These results indicate that, while the S358L mutation has minimal impact on TMEM43's regulatory effect on SREBP, it may compromise TMEM43 protein stability. Integrating the insights from our current investigation, these collective observations lead us to postulate that the mutation-induced reduction of TMEM43 expression prompts the activation of SREBPs, thereby leading to the enhanced adipocyte differentiation in ARVC.

**Figure 7:**
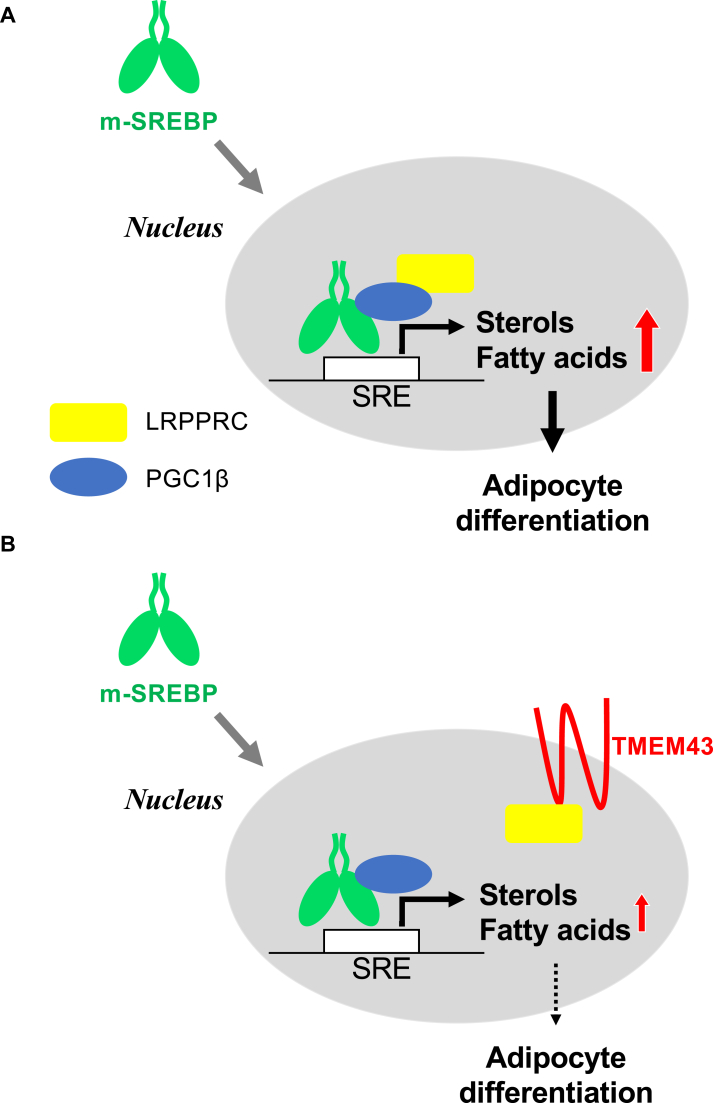
**Mode of action of TMEM43 on the suppression of SREBP**. LRPPRC activates SREBP in cooperation with PGC1β (*A*). If TMEM43 is overexpressed on nuclear membrane, LRPPRC is recruited to TMEM43 and SREBP activity is suppressed (*B*). By this mechanism, TMEM43 suppresses adipocyte differentiation. (m-SREBP: mature form of SREBP).

Our findings suggest that TMEM43 downregulates the transcription-stimulating capacity of SREBPs by sequestering the LRPPRC co-activator at the nuclear membrane. The SREBP activity is subject to stringent regulation, encompassing diverse mechanisms and the participation of various signaling proteins. Among the well-documented regulatory mechanisms is the retention of the INSIG-SCAP-SREBP complex at the ER membrane in the presence of sterols (43, 44). Manipulating cellular cholesterol levels also involves mTORC1, which has been shown to suppress cholesterol delivery to lysosomes, consequently lowering the ER cholesterol content and activating SREBPs (45). Beyond the ER membrane and Golgi apparatus, another mechanism entails AMP-activated protein kinase (AMPK), which phosphorylates SREBPs, thereby inhibiting their processing (46). Furthermore, nuclear SREBPs can be subjected to phosphorylation by glycogen synthase kinase3β (GSK3β), leading to their degradation *via* the ubiquitin/proteasome pathway (47). The inhibition of SREBP activation by TMEM43 stands apart as a distinct and unprecedented mechanism, involving the sequestration of its transcriptional co-factor, LRPPRC, at the nuclear membrane, thereby expanding our understanding of the intricate regulatory network governing SREBP activity (Fig. 7).

Mutations in LRPPRC have been identified as a causative factor in Leigh syndrome, French-Canadian type (LSFC), a severe mitochondrial disorder characterized by neurodegeneration and impaired energy metabolism (48, 49). Mitochondrial LRPPRC facilitates oxidative phosphorylation by stabilizing mitochondrial DNA-derived transcripts, thereby ensuring proper expression of mitochondrial genes (50, 51). Whether TMEM43 modulates this mitochondrial function of LRPPRC remains unknown. Furthermore, no clinical link has been reported to date between LSFC caused by LRPPRC mutations and ARVC associated with TMEM43 mutations.

Our work has generated several important questions that will be the basis for further deep investigations. One question pertains to the specificity of LRPPRC-mediated inhibition of SREBPs by TMEM43. LRPPRC belongs to the family of PPR motif-containing proteins, a group recognized for their ability to bind to RNA and regulate gene expression for controlling energy metabolism (33). Importantly, LRPPRC has also been identified as a binding partner for PGC1α and PGC1β, enhancing their co-activator activities (37). Initially characterized as co-activators for peroxisome proliferator-activated receptor γ (PPARγ) (52), the PGC1 family has since been acknowledged for their co-activator roles in the regulation of multiple other transcription factors related to energy metabolism, including forkhead transcription factor-1 (FOXO1) (53), hepatocyte nuclear factor 4 alpha (HNF4a) (54), liver X receptor (LXR) (38), myocyte enhancer factor-2 (MEF-2) (55), PPARα (56), and SREBP (38). Therefore, the sequestration of LRPPRC at the nuclear membrane may have broader implications for the activity of these additional transcriptional factors, mediated through PGC1α and PGC1β. Future work will be critical for a more precise understanding of the intricate roles of LRPPRC in energy metabolism.

A second question is how TMEM43 stabilizes on the nuclear membrane. In our previous work, we demonstrated that the physical association of TMEM43 with SQS stabilizes TMEM43 on the ER membrane, leading to the activation of TGFβ signaling through the stabilization of SMAD4 (11). However, given that SQS is localized exclusively on the ER membrane, the precise mechanisms governing the stabilization of TMEM43 on the nuclear membrane, where it impacts SREBP signaling, remain elusive. Importantly, the S358L mutation in TMEM43 has been shown to reduce its expression on both the ER and nuclear membranes (41). These observations raise the possibility that an alternate mechanism may be responsible for stabilizing TMEM43 on the nuclear membrane. An alternative hypothesis is that TMEM43 could undergo redistribution between the ER and nuclear membranes. Further investigations will be required to unravel the TMEM43 stabilization on the nuclear membrane and its potential dynamic interplay with the ER membrane.

A third question is whether the observed enhancement of adipogenesis following TMEM43 knockdown is mediated solely by the LRPPRC–SREBP pathway. Although our results suggest that this pathway plays a significant role in promoting lipogenic gene expression, adipocyte differentiation is a highly orchestrated process involving multiple transcriptional regulators, signaling cascades, and metabolic cues (39). It remains possible that TMEM43 influences adipogenesis through additional, SREBP-independent mechanisms. Therefore, further investigation is necessary to dissect the relative contribution of the LRPPRC–SREBP pathway to TMEM43-mediated regulation of adipogenesis and to determine whether other parallel pathways are also involved.

In conclusion, the present study illuminates the role of TMEM43 as a negative regulator of SREBP signaling pathway, and underscores its significance in the pathogenesis of ARVC5. The insights gained from this study offer a fresh perspective for the investigation of the SREBP and TGFβ signaling cascades, serving as a foundation for subsequent studies of ARVC.

## Experimental procedures

### Cell culture

A549 cells (ATCC), HEK293 cells (RIKEN Cell Bank) and 3T3-L1 cells (ATCC) were maintained in Dulbecco's modified Eagle Medium (DMEM) supplemented with 100 units/ml of penicillin, 100 μg/ml of streptomycin sulfate and 10% fetal bovine serum (FBS) at 37 °C in a humidified 5% CO_2_ incubator. CHO-K1 cells (RIKEN Cell Bank) were maintained in 1:1 mixture of Ham's F-12 medium and DMEM supplemented with 100 units/ml penicillin, 100 μg/ml streptomycin sulfate and 5% FBS at 37 °C in a humidified 5% CO2 incubator.

### Antibodies

Anti-actin antibody (ab11003) and anti-TMEM43 antibody (ab113116) were purchased from Abcam. Anti-SQS antibody (13128-1-AP), anti-CANX antibody (10427-2-AP), and anti-LRPPRC antibody (21175-1-AP) were obtained from Proteintech. Anti-NUP98 antibody (#2598) was purchased from Cell Signaling Technology. Anti-FLAG antibody (F1804) was from Sigma. Anti-c-Myc antibody (04362–76) was from Nacalai Tesque.

### Plasmids

Cloning of *TMEM43* gene into pCMV-3Tag-9 vector has already been reported (11). *EGFP* gene was amplified from pEGFP-N1 (Clontech) by PCR (Forward primer: TACCGTCGACCTCGAGATGGTGAGCAAGGGCGA, Reverse primer: GTTTCTG CTCCTCGAGCTTGTACAGCTCGTCCA) and was inserted into pCMV-3Tag-9 or pCMV-3Tag-9-TMEM43 at Xho1 site by In-Fusion HD Cloning Kit (Takara Bio). TMEM43 gene was amplified from pCMV-3Tag-9-TMEM43 by PCR (Forward primer: CTGCAGGAATTCGATATGGCCGCGAATTATTCCA, Reverse primer: ATCGATAAGCTTGATCTCCAACTTTTTGGCTGGC) and was subcloned into pCMV-3Tag-3a vector at EcoRV site by In-Fusion HD Cloning Kit. Luciferase gene was amplified from pGL3-Basic (Promega) by PCR (Forward primer: CTGCAGGAATTCGATATGGAAGACGCCAAAAACAT, Reverse primer: ATCGATAAGCTTGATCACGGCGATCTTTCCGCC) and was subcloned into pCMV-3Tag-3a vector at EcoRV site by In-Fusion HD Cloning Kit. LRPPRC gene was amplified from HEK293 cDNA library by PCR (Forward primer: CTGCAGGAATTCGATATGGCAGCCCTGCTGAGA, Reverse primer: ATCGATAAGCTTGATAGAAGAGTTTTCCCTCAATTT) and was cloned into pCMV-3Tag-9 vector at EcoRV site by In-Fusion HD Cloning Kit. Then, LRPPRC gene was amplified from pCMV-3Tag-9-LRPPRC by PCR with the same primer set and was subcloned into pCMV-3Tag-3a vector at EcoRV site by In-Fusion HD Cloning Kit. PGC1β gene was amplified from HEK293 cDNA library by PCR (Forward primer: CTGCAGGAATTCGATATGGCGGGGAACGACTGC, Reverse primer: ATCGATAAGCTTGATATGCAGGCTCTGCTGGGC) and was cloned into pCMV-3Tag-9 vector at EcoRV site by In-Fusion HD Cloning Kit. Then, PGC1β gene was amplified from pCMV-3Tag-9-PGC1β by PCR with the same primer set and was subcloned into pCMV-3Tag-3a vector at EcoRV site by In-Fusion HD Cloning Kit. Mature SREBP1a(1–490) gene was amplified from BxPC-3 cDNA library by PCR (Forward primer: CGGGCTGCAGGAATTCATGGACGAGCCACCCTTCA, Reverse primer: GCTTGATATCGAATTCTACAGGGCCAGGCGGGA) and was cloned into pCMV-3Tag-1a at EcoR1 site by In-Fusion HD Cloning Kit.

### Western blotting

The harvested cells were lysed by sonication in a lysis buffer (50 mM Tris-HCl (pH 7.5), 120 mM NaCl, 5 mM EDTA (pH 8.0), and 0.5% Nonidet P-40) containing a protease inhibitor cocktail (Nacalai Tesque). The lysates were centrifuged at 20,000 g for 15 min, and the concentration of the protein in each lysate was determined by a BCA protein assay kit (Thermo Fisher Scientific). The lysates were mixed with an SDS-PAGE loading buffer and boiled for 3 min. The proteins in the sample were separated by SDS-polyacrylamide gel electrophoresis (SDS-PAGE) and transferred to a nitrocellulose membrane (GE Healthcare Life Sciences) by electroblotting. After incubation with primary and secondary antibodies, the proteins were detected using an ECL Prime Western Blotting Detection Reagent (GE Healthcare Life Sciences). The resulting luminescence was analyzed with an ImageQuant LAS 500 (GE Healthcare Life Sciences).

### Real-time quantitative PCR

A549 cells (2.5 × 10^4^) were seeded onto a 12-well plate. Next day, siEGFP (Ambion, AM4626), siSQS (5′-GCCACUUUGGCUGCCUGUUAUAAUA-3′ and 5′-AUUAUAACAGGCAGCCAAAGUGGC-3′) or siTMEM43 (5′-CCCGGAGAGAACAUGUCAAAGUUAA-3′ and 5′-UUAACUUUGACAUGUUCUCUCCGGG-3′) was transfected using Lipofectamine RNAiMAX Transfection Reagent (Thermo Fisher Scientific). After 4 days, total RNA was isolated with ISOGEN (NIPPON GENE) following the manufacturer's protocol. cDNA was synthesized with Primescript first strand synthesis kit (Takara Bio). For qPCR analysis, cDNA was added to a solution containing the primers (500 nM each) and Fast SYBR Green Reagent (Thermo Fisher Scientific), and the reaction (in triplicates) was run in 7500 Fast Real Time PCR System (Applied Biosystems). The sequences of primers for the respective genes are as follow. TMEM43 (Forward): AGCAAAAACTTCGACCGAGA. TMEM43 (Reverse): CTG CCAATTTGGACAAAGG. SQS (Forward): GGTGATGCCCAAGATGGA. SQS (Reverse): TGGTCTGATTGAGATACTTGTAGCA. HMGCR (Forward): GCCGACAGTTCTGAACTGGAACA. HMGCR (Reverse): GAACCTGAGACCTCTCTGAAAGAG. INSIG1 (Forward): AAGACTTCAGGGTAAGTCATCA. INSIG1 (Reverse): CGTGTATAATGGTGTCTATCAG. FAS (Forward): GCAAATTCGACCTTTCTCCAGAA. FAS (Reverse): GTAGGACCCCGTGGAATGTC. β-actin (Forward): GCAAAGACCTGTACGCCAACA. β-actin (Reverse): TGCATC CTGTCGGCAATG. Data were analyzed by ΔΔ-Ct method with β-actin as reference control.

### SRE reporter assay

HEK293 cells (9.5 × 10^3^) were seeded onto a 96-well plate and incubated for 24 h. Then, the cells were co-transfected with pCMV-3Tag-9, pCMV-3Tag-9-TMEM43, or pCMV-INSIG1-FLAG together with an SRE-1-driven luciferase reporter plasmid (pSRE-Luc) and a β-galactosidase reporter plasmid (pAc-β-gal) using FuGENE HD Transfection Reagent (Promega). After 24 h, the cells were treated with 50 μM of compactin (Tokyo Chemical Industry Co) for 24 h. Afterward, the cells in each well were lysed by freeze-thaw with Reporter Lysis Buffer (Promega), and aliquots were used to measure luciferase and β-galactosidase activities. Luciferase activity was measured using the Steady-Glo Luciferase Assay System (Promega), and β-galactosidase activity was measured using the β-galactosidase Enzyme Assay System (Promega). Luciferase activity was normalized to β-galactosidase activity. To monitor the activity of the mature form of SREBP2 or SREBP1a, we transfected HEK293 cells with pcDNA-FLAG-SREBP2 (mature) or pCMV-3Tag-1a-SREBP1a (mature) together with other plasmids. After 48 h, luciferase activity was measured.

To investigate the activity of TMEM43 against SREBP in CHO-K1 cells, cells (8 × 10^3^) were seeded onto a 96-well plate and incubated for 24 h. Then, the cells were co-transfected with pCMV-3Tag-9-EGFP or pCMV-3Tag-9-TMEM43-EGFP together with pSRE-Luc and pAc-β-gal using FuGENE HD Transfection Reagent (Promega). After 24 h, the medium was changed to the medium containing 5% FBS (+lipids) or the medium containing 5% lipoprotein-deficient serum (LPDS) and 50 μM of compactin (−lipids) for 24 h. After that, luciferase activity was measured.

### Fluorescent microscopy

To investigate the co-localization of TMEM43 and CANX, NUP98, or LRPPRC, CHO-K1 cells (1 × 10^4^) were seeded onto a 35 mm dish (4 compartment). Then, the cells were transfected with pCMV-3Tag-9-EGFP or pCMV-3Tag-9-TMEM43-EGFP using FuGENE HD Transfection Reagent. After 48 h, cells were fixed with 4% paraformaldehyde for 15 min. Cells were then washed with PBS and permeabilized with 0.5% TritonX-100/PBS. After 15 min, cells were washed with PBS and incubated with 3% BSA/PBS at 4 °C overnight. Then, cells were washed with PBS and were stained with anti-CANX antibody (1:200), anti-NUP98 antibody (1:200), or anti-LRPPRC antibody (1:200). After 1 h, cells were washed with PBS and were stained with goat anti-rabbit IgG H&L (Alexa Fluor 568) (abcam, ab175471) for 1 h in the dark. After that, cells were washed with PBS, and the fluorescence signals were visualized with a CV-1000 confocal microscope (Yokogawa).

To investigate the localization of LRPPRC in cells with TMEM43 knockdown, A549 cells (1.25 × 10^4^) were seeded onto a 35 mm dish (4 compartment). Then, siEGFP or siTMEM43 was transfected using Lipofectamine RNAiMAX Transfection Reagent (Thermo Fisher Scientific). After 4 days, cells were fixed, permeabilized, and stained with α-LRPPRC antibody and α-Rabbit IgG H&L (Alexa Fluor 568). Alexa Fluor 568-bound LRPPRC was visualized by fluorescence microscopy.

### Fractionation of nucleus and cytoplasm

HEK293 cells (1.4 × 10^6^) were seeded onto a 10 cm dish and incubated for 24 h. Then, the cells were transfected with pCMV-3Tag-9 or pCMV-3Tag-9-TMEM43 together with pcDNA-FLAG-SREBP2 (mature) using FuGENE HD Transfection Reagent. After 24 h, the cells were collected and fractionated into a nuclear or cytoplasmic fraction by Nuclear/Cytosolic Fractionation Kit (Cell Biolabs) following the manufacturer's protocol. Each fraction was analyzed by immunoblotting.

### Immunoprecipitation

HEK293 cells (1.4 × 10^6^) were seeded onto a 10 cm dish. The next day, the cells were transfected with plasmids using FuGENE HD Transfection Reagent and incubated for 24 h. Collected cells were lysed in a lysis buffer containing a protease inhibitor cocktail with sonication. The lysates were centrifuged at 20,000 g for 15 min, and the concentration of the protein in each lysate was determined by a BCA protein assay kit. The lysates were pre-cleared with Protein G Sepharose 4 Fast Flow (GE Healthcare) at 4 °C for 1 h and mixed with α-FLAG antibody at 4 °C for 1 h. Then, the immunocomplex was captured by Protein G Sepharose 4 Fast Flow at 4 °C for 1 h. After washing protein G-bound immunocomplex with lysis buffer three times, bound proteins were eluted in lysis buffer containing 100 μg/ml of FLAG peptide (Sigma-Aldrich). Proteins were separated by SDS-PAGE and detected by silver stain. The ∼130 kDa protein co-precipitated with TMEM43-3 × FLAG was cut off and identified by Mass analysis (RIKEN). When Myc-tagged protein was immunoprecipitated, anti-c-Myc antibody-conjugated agarose beads (Nacalai) were mixed with the lysates at 4 °C for 2 h. After washing the immunocomplex with lysis buffer three times, the bound proteins were eluted in lysis buffer containing 100 μg/ml of c-Myc peptide (Sigma-Aldrich). The eluted proteins were also analyzed by immunoblotting.

### Adipocyte differentiation of 3T3-L1 cells

3T3-L1 cells (5 × 10^4^) were seeded onto a 12-wells plate. Next day, siEGFP, siTMEM43#1 (5′-AGCUGAGGAGGUGUUUCGUAGAGAA-3′and 5′-UUCUCUACGAAACACCUCCUCAGCU-3′), siTMEM43#2 (5′-GGAUGGCCAUGUUUAUGGGCCUCAA-3′ and 5′-UUGA GGCCCAUAAACAUGGCCAUCC) or siTMEM43#3 (5′-ACGAGGUGACCAACUAAUCCCAUAU-3′ and 5′-AUAUGGGAUUAGUUGGUCACCUGGU-3′) was transfected using Lipofectamine RNAiMAX Transfection Reagent. After 2 days, the medium was replaced with MDI medium (DMEM supplemented with 100 units/ml of penicillin, 100 μg/ml of streptomycin sulfate, 10% FBS, 10 μg/ml of insulin, 1 μM of dexamethasone, and 0.5 mM of isobutylmethylxanthine) and incubated for 3 days. Then, the medium was replaced with insulin medium (DMEM supplemented with 100 units/ml of penicillin, 100 μg/ml of streptomycin sulfate, 10% FBS, and 10 μg/ml of insulin) and incubated for 3 days. After then, the medium was replaced with a normal medium and incubated for additional 3 days. Adipocyte-differentiated cells were stained with Oil red O (Sigma-Aldrich). Oil Red O incorporated into the cells was extracted with 2-propanol, and the absorbance (492 nm) was measured.

## Data availability

All data are contained within the manuscript.

## Supporting information

This article contains supporting information.

## Conflict of interest

The authors declare that they have no conflicts of interest with the contents of this article.
