# Supplementary material for: Suppression of SREBP by a transmembrane protein mutated in cardiomyopathy
Source: J Biol Chem. 2025 Aug 28;301(10):110644. doi: 10.1016/j.jbc.2025.110644 (PMC12494557; doi:10.1016/j.jbc.2025.110644)
Supplement: Supplemental Information [file mmc1.pdf]

Supplemental Information for

Suppression of SREBP by a Transmembrane Protein Mutated in Cardiomyopathy

Yasushi Takemoto[1,4,5,*], Manchir Tserendagva[1,5], Motonari Uesugi[1,2,3,*]

[1] Institute for Chemical Research, Kyoto University, Uji, Kyoto 611-0011, Japan

[2] Institute for Integrated Cell-Material Sciences (WPI-iCeMS), Kyoto University, Uji, Kyoto 611-0011, Japan

[3] School of Pharmacy, Fudan University, Shanghai 201203, China

[4] Present address: Department of Radiation Oncology, Fukushima Medical University School of Medicine, 1 Hikarigaoka, Fukushima, Fukushima 960-1295, Japan

[5] These authors contributed equally

*Corresponding authors: uesugi@scl.kyoto-u.ac.jp and takemotoyasushi@gmail.com

Table of contents

Supplemental figures ··S2-S10

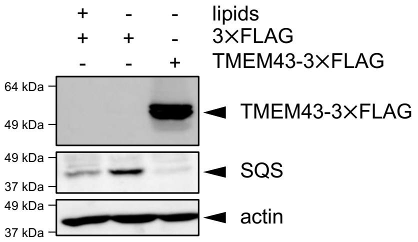

Figure S1. Lipid depletion-induced SQS expression is suppressed by TMEM43.
Effect of TMEM43 on lipid depletion-induced SQS expression. CHO-K1 cells were transfected with pCMV-3Tag-3a (2 µg) or pCMV-3Tag-3a-TMEM43 (2 µg) for 24 h. Then, the cells were cultured in the medium containing FBS5% (+lipids) or LPDS5% plus 50 µM of compactin (-lipids) for 24 h. The lysates were analyzed by western blotting. Similar results were obtained from two independent experiments.

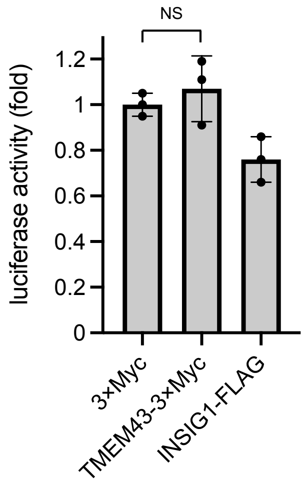

Figure S2. TMEM43 has no detectable effect on CMV promoter-controlled luciferase activity.
Effect of TMEM43 on luciferase activity. HEK293 cells were transfected with pCMV-3Tag-9 (0.05 µg), pCMV-3Tag-9-TMEM43 (0.05 µg) or pCMV-INSIG1-FLAG (0.05 µg) together with pCMV-3Tag-3a-luciferase (0.045 µg) and pAc-β-gal (0.005 µg). After 48 h, luciferase activity was measured (n = 3). Data are presented as mean of ± SD. Statistical significance was determined by paired two-tailed Student's *t* test. NS : not significant. Similar results were obtained from two independent experiments.

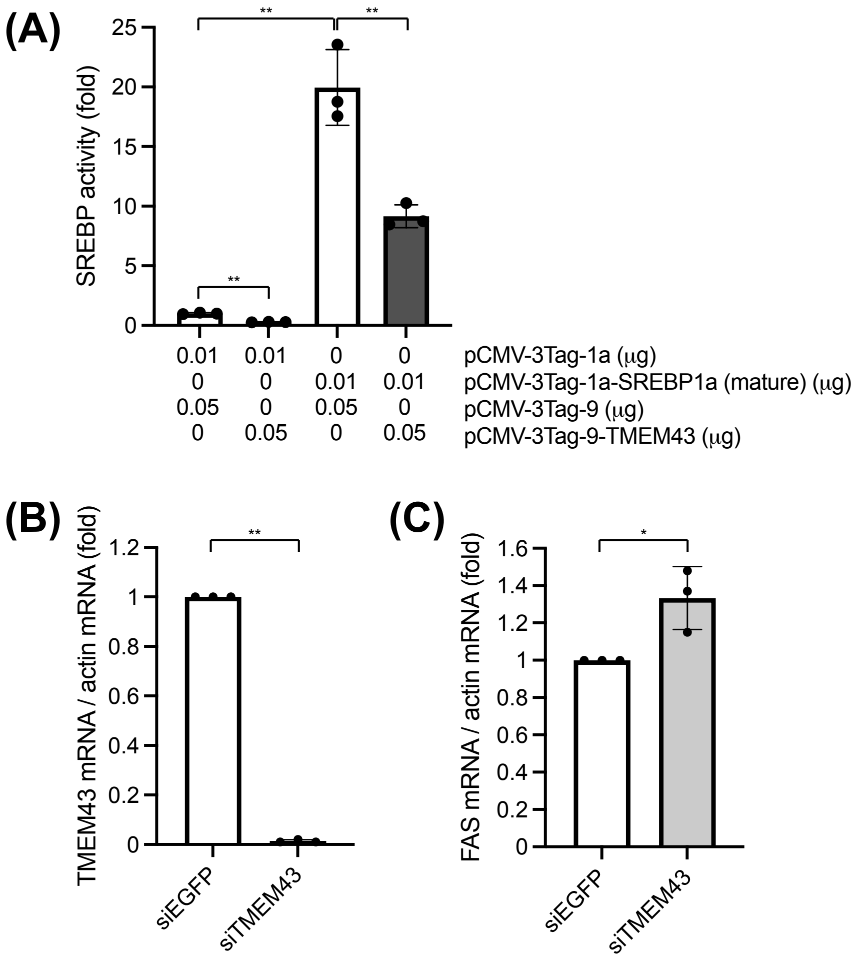

Figure S3. TMEM43 suppresses SREBP1a transcriptional activity and FAS expression. (A) Effect of TMEM43 on SREBP1a transcriptional activity. HEK293 cells were transfected with pCMV-3Tag-9 (0.05 µg) or pCMV-3Tag-9-TMEM43 (0.05 µg), pCMV-3Tag-1a-SREBP1a (mature) (0.01 µg) or pCMV-3Tag-1a (0.01 µg) together with pSRE-Luc (0.035 µg) and pAc-β-gal (0.005 µg). After 48 h, luciferase activity was measured (n = 3). Data are presented as mean of ± SD. Statistical significance was determined by paired two-tailed Student's *t* test. **$p < 0.01$. Similar results were obtained from three independent experiments. (B and C) TMEM43 knockdown increases FAS gene expression. A549 cells were transfected with each siRNA (siEGFP or siTMEM43) and incubated for 4 days. The mRNA level of TMEM43 (B) or FAS (C) was assessed by qPCR analysis. Data were analyzed by ΔΔCt method with actin as reference control (n = 3). Data are presented as mean of ± SD. Statistical significance was determined by paired two-tailed Student's *t* test. *$p < 0.05$, **$p < 0.01$.

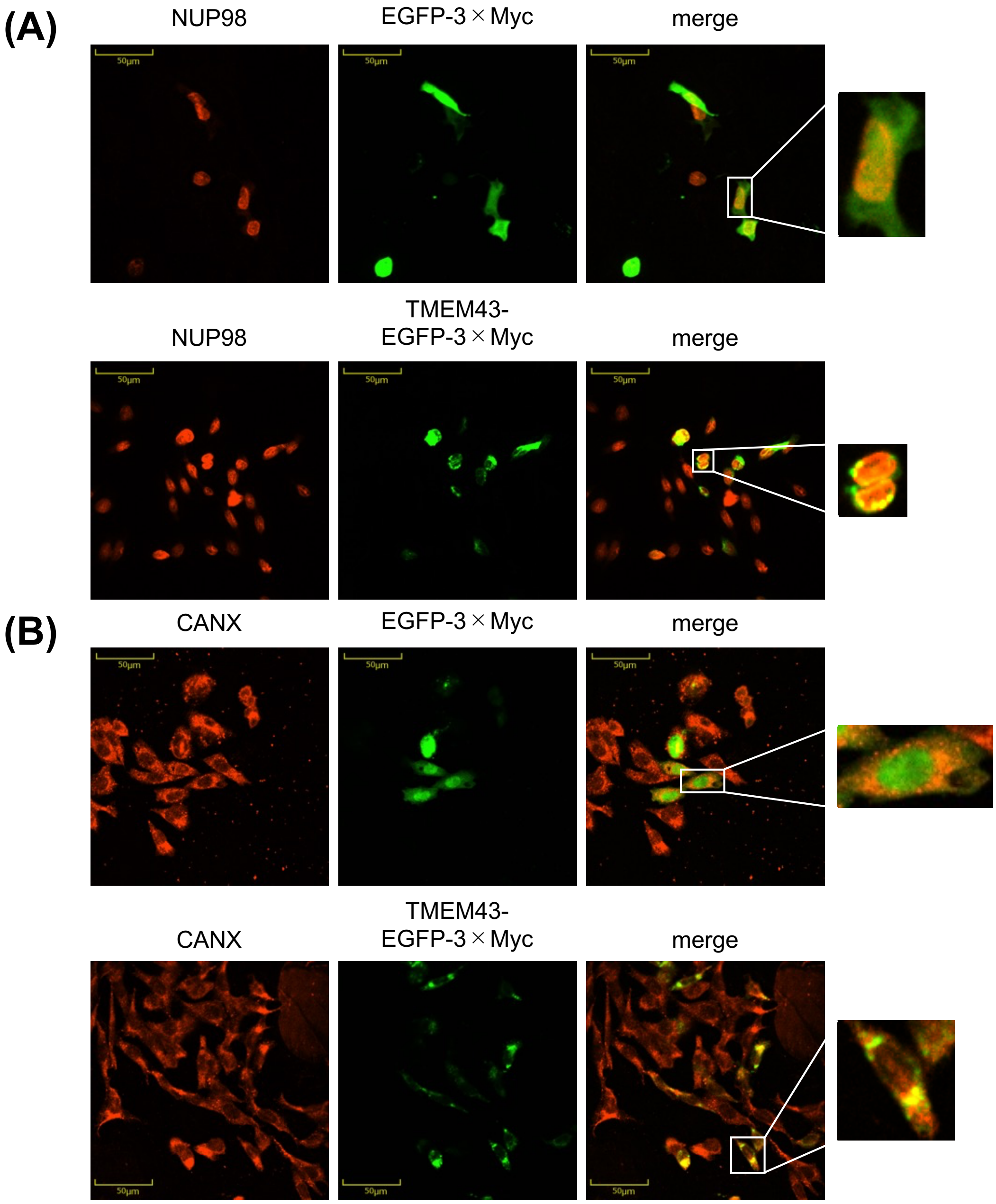

Figure S4. Localization of TMEM43-EGFP-3×Myc.
Fluorescence imaging. CHO-K1 cells were transfected with pCMV-3Tag-9-EGFP or pCMV-3Tag-9-TMEM43-EGFP. After 24 h, cells were fixed, permeabilized and stained with α-NUP98 antibody (A) or α-CANX antibody (B) and α-Rabbit IgG H&L (Alexa Fluor® 568). EGFP-fused proteins and Alexa Fluor® 568-bound NUP98 or CANX were visualized by fluorescence microscopy. Scale bar : 50 μm. Similar results were obtained from two independent experiments.

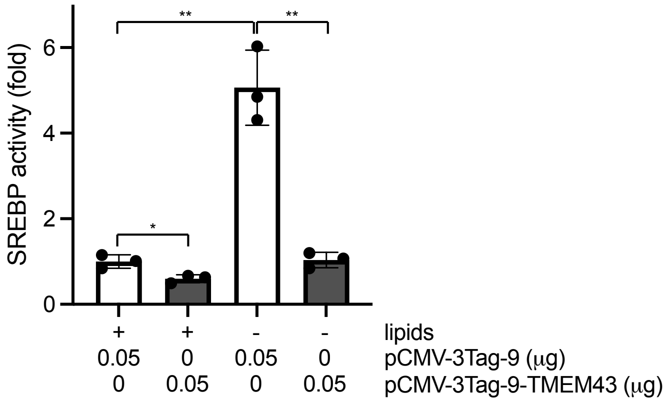

Figure S5. Lipid depletion-induced SREBP activation is suppressed by EGFP-fused TMEM43.
Effect of TMEM43-EGFP-3 × Myc on SREBP transcriptional activity. CHO-K1 cells were transfected with pCMV-3Tag-9-EGFP (0.05 μg) or pCMV-3Tag-9-TMEM43-EGFP (0.05 μg) together with pSRE-Luc (0.045 μg) and pAc-β-gal (0.005 μg) and incubated for 24 h. Then, the medium was replaced with the medium containing 5% FBS (+lipids) or the medium containing 5% LPDS plus 50 μM of compactin (-lipids). After 24 h, luciferase activity was measured (n = 3). Data are presented as mean of ± SD. Statistical significance was determined by paired two-tailed Student's *t* test. *$p < 0.05$, **$p < 0.01$. Similar results were obtained from two independent experiments.

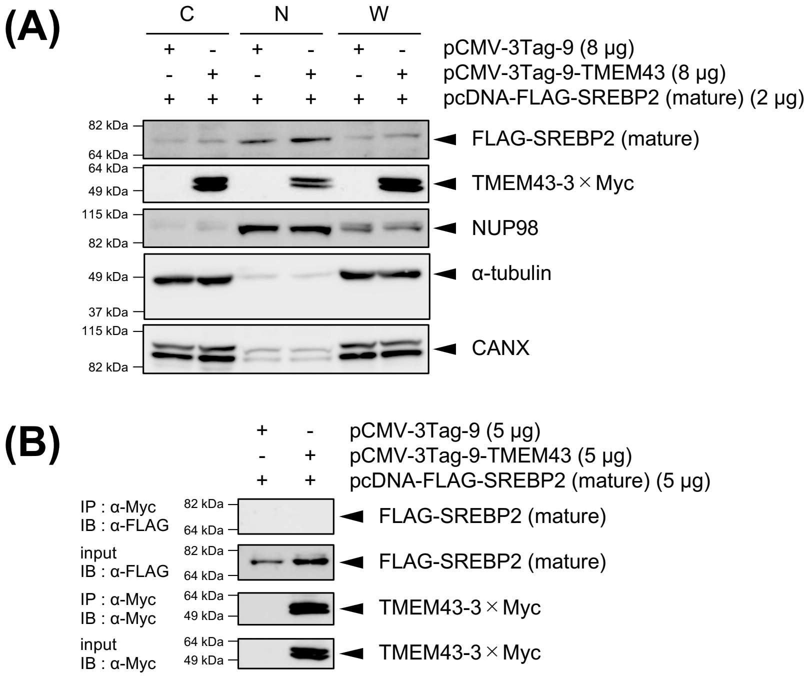

Figure S6. TMEM43 fails to inhibit the translocation of mature SREBP2 into the nucleus. (A) Effect of TMEM43 expression on the translocation of mature SREBP2 into the nucleus. HEK293 cells were transfected with pCMV-3Tag-9 (8 μg) or pCMV-3Tag-9-TMEM43 (8 μg) together with pcDNA-FLAG-SREBP2 (mature) (2 μg). After 24 h, cells were fractionated into the cytosol fraction and the nuclear fraction. The same weight of lysate (22.1 μg) was analyzed by western blotting. C means the cytosol fraction, N means the nuclear fraction and W means the whole cell lysate. Similar results were obtained from two independent experiments.
(B) TMEM43 dose not bind to mature SREBP2. HEK293 cells were expressed with 3 × Myc or TMEM43-3 × Myc and FLAG-SREBP2 (mature). After immunoprecipitation with α–Myc antibody, eluted proteins were analyzed by western blotting. Similar results were obtained from two independent experiments.

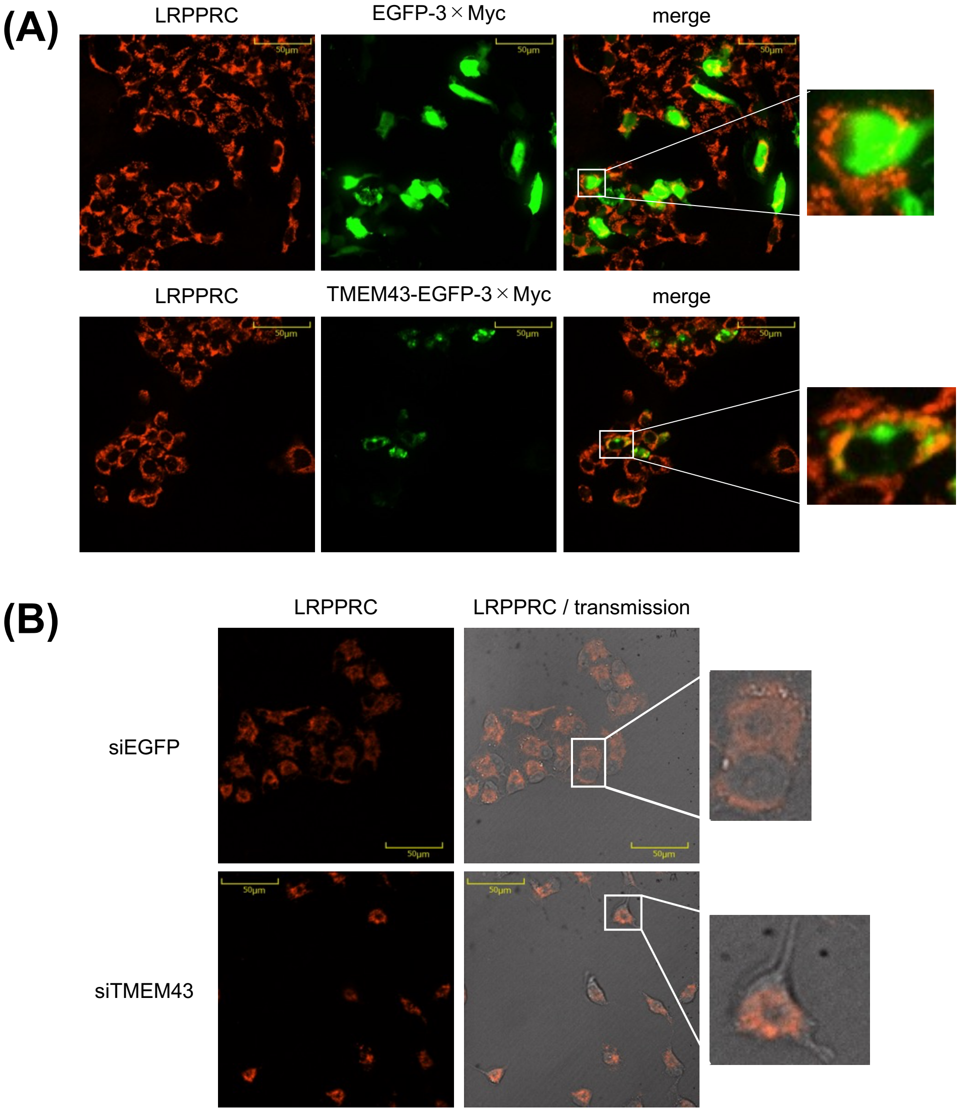

Figure S7. Co-localization of LRPPRC and TMEM43-EGFP-3×Myc.
Fluorescence imaging. (A) CHO-K1 cells were transfected with pCMV-3Tag-9-EGFP (top) or pCMV-3Tag-9-TMEM43-EGFP (bottom). After 48 h, cells were fixed, permeabilized and stained with α-LRPPRC antibody and α-Rabbit IgG H&L (Alexa Fluor® 568). EGFP-fused proteins and Alexa Fluor® 568-bound LRPPRC were visualized by fluorescence microscopy. Scale bar : 50 μm. Similar results were obtained from two independent experiments. (B) A549 cells were transfected with each siRNA (siEGFP or siTMEM43). After 4 days, cells were fixed, permeabilized and stained with α-LRPPRC antibody and α-Rabbit IgG H&L (Alexa Fluor® 568). Alexa Fluor® 568-bound LRPPRC were visualized by fluorescence microscopy. Scale bar : 50 μm. Similar results were obtained from three independent experiments.

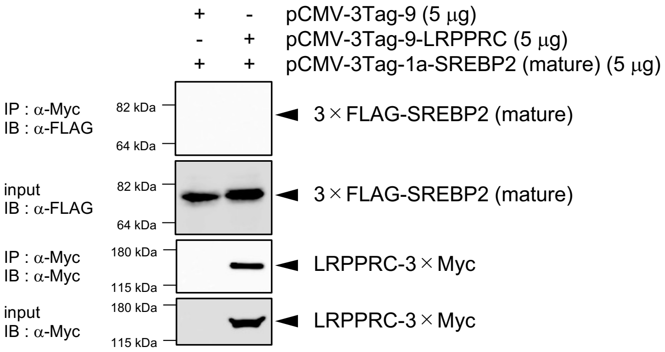

Figure S8. LRPPRC fails to bind to mature SREBP2.
LRPPRC dose not bind to mature SREBP2. HEK293 cells were expressed with 3×Myc or LRPPRC-3×Myc and 3×FLAG-SREBP2 (mature). After immunoprecipitation with α–Myc antibody, eluted proteins were analyzed by western blotting. Similar results were obtained from two independent experiments.

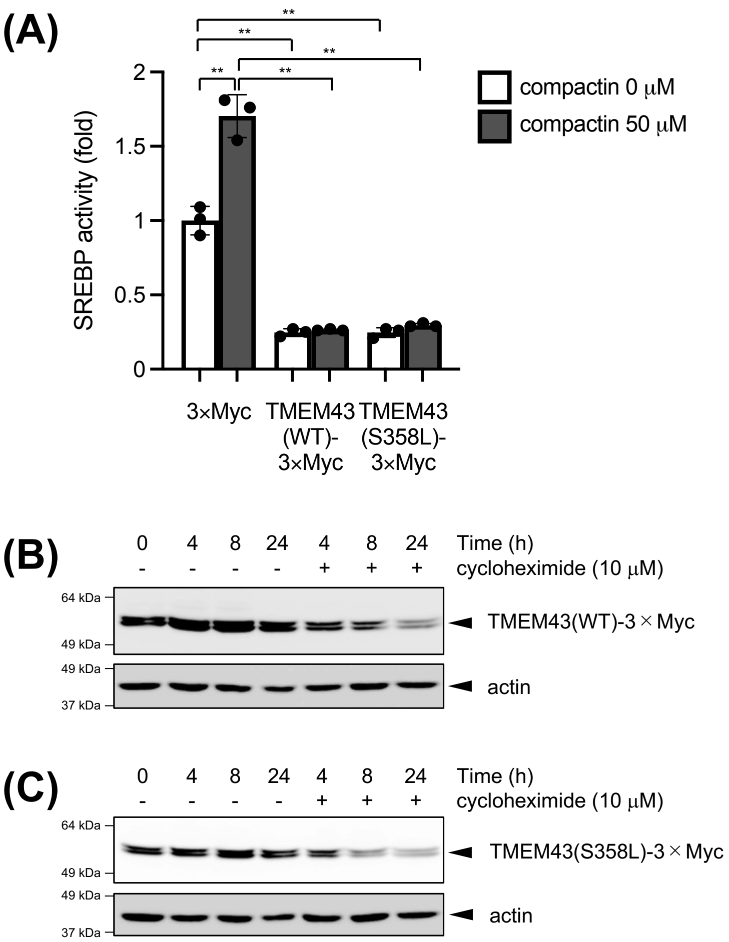

Figure S9. Comparison of TMEM43 wild type and S358L mutant.
(A) Effect of TMEM43 wild type (WT) and S358L mutant on SREBP transcriptional activity. HEK293 cells were transfected with pCMV-3Tag-9 (0.05 μg), pCMV-3Tag-9-TMEM43(WT) (0.05 μg) or pCMV-3Tag-9-TMEM43(S358L) (0.05 μg) together with pSRE-Luc (0.045 μg) and pAc-β-gal (0.005 μg) and incubated for 24 h. Then, DMSO or compactin (50 μM) was treated. After 24 h, luciferase activity was measured (n = 3). Data are presented as mean of ± SD. Statistical significance was determined by paired two-tailed Student's *t* test. $^{**}p < 0.01$. Similar results were obtained from two independent experiments. (B) Comparison of protein stability of TMEM43(WT) and S358L mutant. HEK293 cells were transfected with pCMV-3Tag-9-TMEM43(WT) or pCMV-3Tag-9-TMEM43(S358L) and incubated for 24 h. Then, DMSO or cycloheximide (10 μM) was treated. After each indicated time, cells were collected. The lysates were analyzed by western blotting. Similar results were obtained from two independent experiments.
